# Key genes involved in nonalcoholic steatohepatitis improvement after bariatric surgery

**DOI:** 10.3389/fendo.2024.1338889

**Published:** 2024-02-26

**Authors:** Xiyu Chen, Shi-Zhou Deng, Yuze Sun, Yunhu Bai, Yayun Wang, Yanling Yang

**Affiliations:** ^1^Department of Hepatobiliary Surgery, Xi-Jing Hospital, The Fourth Military Medical University, Xi’an, China; ^2^Department of General Surgery, 988 Hospital of Joint Logistic Support Force, Zhengzhou, China; ^3^Specific Lab for Mitochondrial Plasticity Underlying Nervous System Diseases, National Demonstration Center for Experimental Preclinical Medicine Education, The Fourth Military Medical University, Xi’an, China

**Keywords:** bariatric surgery, sleeve gastrectomy, nonalcoholic fatty liver disease, nonalcoholic steatohepatitis, gene expression omnibus datasets, competitive endogenous RNA

## Abstract

**Background:**

Nonalcoholic steatohepatitis (NASH) is the advanced stage of nonalcoholic fatty liver disease (NAFLD), one of the most prevalent chronic liver diseases. The effectiveness of bariatric surgery in treating NASH and preventing or even reversing liver fibrosis has been demonstrated in numerous clinical studies, but the underlying mechanisms and crucial variables remain unknown.

**Methods:**

Using the GSE135251 dataset, we examined the gene expression levels of NASH and healthy livers. Then, the differentially expressed genes (DEGs) of patients with NASH, at baseline and one year after bariatric surgery, were identified in GSE83452. We overlapped the hub genes performed by protein-protein interaction (PPI) networks and DEGs with different expression trends in both datasets to obtain key genes. Genomic enrichment analysis (GSEA) and genomic variation analysis (GSVA) were performed to search for signaling pathways of key genes. Meanwhile, key molecules that regulate the key genes are found through the construction of the ceRNA network. NASH mice were induced by a high-fat diet (HFD) and underwent sleeve gastrectomy (SG). We then cross-linked the DEGs in clinical and animal samples using quantitative polymerase chain reaction (qPCR) and validated the key genes.

**Results:**

Seven key genes (FASN, SCD, CD68, HMGCS1, SQLE, CXCL10, IGF1) with different expression trends in GSE135251 and GSE83452 were obtained with the top 30 hub genes selected by PPI. The expression of seven key genes in mice after SG was validated by qPCR. Combined with the qPCR results from NASH mice, the four genes FASN, SCD, HMGCS1, and CXCL10 are consistent with the biological analysis. The GSEA results showed that the ‘cholesterol homeostasis’ pathway was enriched in the FASN, SCD, HMGCS1, and SQLE high-expression groups. The high-expression groups of CD68 and CXCL10 were extremely enriched in inflammation-related pathways. The construction of the ceRNA network obtained microRNAs and ceRNAs that can regulate seven key genes expression.

**Conclusion:**

In summary, this study contributes to our understanding of the mechanisms by which bariatric surgery improves NASH, and to the development of potential biomarkers for the treatment of NASH.

## 1 Introduction

Nonalcoholic fatty liver disease (NAFLD) is the most prevalent chronic liver disease (1). It is estimated that more than one billion people worldwide suffer from NAFLD, representing approximately 25% of the global population (2). Nonalcoholic steatohepatitis (NASH), and nonalcoholic fatty liver disease (NAFL), commonly referred to as simple fatty liver, are two of the diseases that fall under the umbrella of NAFLD (3). A patient is considered to have NAFLD if the liver steatosis is more than 5%. If the steatosis is accompanied by hepatocellular balloon degeneration and lobular inflammation, the patient is considered to have NASH (4). Patients with NAFLD are often associated with metabolic syndrome comorbidities such as obesity, hyperlipidemia, hypertension, and type 2 diabetes mellitus (T2DM) (5). They share the same epidemiologic and pathophysiologic features (6). T2DM seems to be the most significant risk factor for NAFLD and NASH among these comorbidities, as well as the most significant predictor of unfavorable outcomes like advanced liver fibrosis and mortality (7).

Although NAFLD is usually clinically asymptomatic, over time NASH can progressively deteriorate and lead to cirrhosis, hepatocellular carcinoma (HCC), end-stage liver disease, or the necessity for transplantation (8). NAFLD treatment currently lacks U.S. Food and Drug Administration (FDA)-approved drugs. Moderate weight loss has demonstrated efficacy in reducing hepatic steatosis, improving the histological manifestations of steatohepatitis, and reversing biopsy-proven fibrosis (9). However, achieving and maintaining the necessary level of weight loss through dietary control and increased physical activity remains challenging for NASH improvement. Right now, bariatric surgery is the most successful therapeutic approach to achieve significant (i.e. 25 - 30%) and enduring weight reduction (10), effectively treating NAFLD (11), along with comorbidities such as Obstructive sleep apnea (OSA) (12), T2DM, and hypertension (13). Bariatric surgery encompasses gastric restriction techniques (e.g. SG), malabsorption procedures (e.g. Biliopancreatic diversion), or a combination thereof (e.g. Roux-en-Y gastric bypass) (14). At present, SG is the most widely utilized bariatric surgery technique globally (15).

The mechanism of NAFLD progression is often described by the “multiple hit” theory, which states that triglyceride accumulation, endoplasmic reticulum stress response, protein misfolding, oxidative stress, and mitochondrial damage in stressed cells lead to a persistent chronic inflammatory state. This leads to excessive activation of immunity and inflammation in liver tissue (16). Activation of the innate and adaptive immune systems triggers, and exacerbates liver inflammation and damage, contributing to NAFLD/NASH (17). Moreover, it has been noticed that an understanding of the interrelationships between gut hormones, the microbiome, obesity, and bariatric surgery may lead to the exploration of new, more specific, non-surgical therapeutic measures to cure severe obesity and its co-morbidities (18). In addition to this, there are a number of factors that have been associated with remission after NASH, including bile acid metabolism (19), gut microbiota (20), gut - brain - liver axis (21), mitochondrial function (22), lipid metabolism (23), and chronic inflammation (24). Future research is needed to explore the effects of these factors on the improvement of obesity-related co-morbidities.

Transcriptome analysis, a widely utilized bioinformatics tool, enables the identification and quantification of transcript levels in different states. It has been extensively employed in mining transcriptome data, elucidating disease pathogenesis, and identifying key targets for diagnosis and treatment (25, 26). The application of transcriptome technology facilitates a better understanding of disease pathogenesis and establishes the connections between RNAs and diseases in the field of disease research.

In this study, we aimed to explore the potential mechanism underlying the improvement of NASH induced by bariatric surgery. We conducted a comprehensive analysis of the differentially expressed genes (DEGs) in NASH patients compared to healthy individuals, as well as before and after bariatric surgery in NASH patients. This allowed us to describe the changes occurring in NASH patients and evaluate the effects of bariatric surgery on NASH. By overlapping the genes with different expression from both datasets with the top 30 genes from protein-protein interaction (PPI) analysis, we identified core genes that play crucial roles in NASH pathogenesis. Furthermore, we employed Genomic enrichment analysis (GSEA) and genomic variation analysis (GSVA) to identify signaling pathways associated with these key genes. To explore regulatory molecules targeting key genes, we constructed a competitive endogenous RNA (ceRNA) network. In addition, we established high fat diet (HFD) induced NASH mouse models and performed Sleeve gastrectomy (SG) to verify the glycolipid metabolism and the expression of key genes following bariatric surgery. Our findings aim to uncover key factors involved in improving NASH through bariatric surgery and provide insights for non-operative treatment strategies for this condition.

## 2 Methods

### 2.1 Bioinformatics analysis

#### 2.1.1 Data source and analysis

The RNA-seq data and corresponding clinical and pathological data used in our study were obtained from the Gene Expression Omnibus (GEO, https://www.ncbi.nlm.nih.gov/geo/) database, including GSE135251, GSE83452, GSE48452, and GSE61260. GSE135251 collected 155 NASH liver samples and 10 healthy liver samples. GSE61260 collected 24 NASH liver samples and 38 healthy liver samples. GSE48452 collected 17 NASH liver samples and 12 healthy liver samples. GSE83452 is a large dataset from liver samples of obese patients, we selected samples for 14 NASH patients, they were diagnosed with “not NASH” 1 year after bariatric surgery. The differentially expressed genes (DEGs) between NASH and normal liver samples in GSE135251 were identified by the “DESeq2” package, while the DEGs between Pre-surgery and Post-surgery in GSE83452 were identified by the “Limma” package (27, 28). The inclusion criteria of DEGs were|log2 FC| ≥ 0.585 and p-adjust < 0.05. Venn diagraming was used to find the overlap DEGs between GSE135251 and GSE83452.

#### 2.1.2 Functional enrichment analysis

To investigate the biological role of DEGs, enrichment analysis using the GO (Gene Ontology) and KEGG (Kyoto Encyclopedia of Genes and Genomes) dictionaries was carried out using the R package “clusterProfiler” (29, 30). Pathways with p < 0.05 were considered statistically significant. A part of the results was visualized using the “ggplot2” package.

#### 2.1.3 Protein-protein interaction network construction

To predict the interaction of DEGs, we construct a protein-protein interaction (PPI) network by an online tool STRING (http://www.string-db.org/) with the cut-off standard as a combined score >0.4. Cytoscape software (version 3.9.0) was used to visualize the PPI network of the DEGs which are linked to each other (31). In addition, to explore the hub genes in the PPI network, we employed a plug-in of Cytoscape named Cytohubba to construct a sub-network by the Maximal Clique Centrality (MCC) algorithm (32).

#### 2.1.4 Gene set enrichment analysis and gene set variation analysis

Depending on the median hub gene value, patients were divided into high and low subgroups. The potential biological significance of the hub genes was investigated using gene set enrichment analysis (GSEA) and gene set variation analysis (GSVA).

Two predefined gene sets including “h.all.v2023.1.Hs.symbols” and “c2.cp.kegg.v2023.1.Hs.symbol” were downloaded on Molecular Signatures Database (MSigDB, https://www.gsea-msigdb.org/gsea/msigdb/index.jsp).

The R package “clusterProfiler” was used to conduct GSEA analysis, the inclusion criteria of the pathway were normalized enrichment score (NES) > 1, adjusted p value < 0.05, and false discovery rate (FDR) < 0.25 (33).

GSVA analysis was performed to calculate the specific pathway scores (GSVA scores) of each sample. Then, the GSVA scores were compared between high and low subgroups (34).

#### 2.1.5 ceRNA network construction

Miranda (http://www.microrna.org/microrna/home.do), miRDB (http://mirdb.org/), and TargetScan Human 7.2 (http://www.targetscan.org/vert_72/) were online databases to predict the miRNAs of mRNA (35–37). SpongeScan was an online database to predict the interaction of lncRNA and miRNA (38). By using the above online tools, we have built the ceRNA networks of hub genes respectively. The ceRNA networks were visualized by Cytoscape software.

### 2.2 Animal experiment

#### 2.2.1 Mouse NASH model

8-week-old male C57BL/6J mice (The Jackson Laboratory, Bar Harbor, Maine, USA) were given a high-fat diet (HFD) with 60% of the kcal derived from fat (D12492, 60% kcal Fat Diet, biopike, China) and the control group was given normal chow diet (NCD), kept on the same diet after the surgery. Mice were housed at 24 - 26 °C with a circadian rhythm of 12 h for ad libitum food and water intake. All animal protocols followed the Guidelines for the Care and Use of Laboratory Animals (license number IACUC-20190107) and were carried out in accordance with the rules of the Animal Welfare Ethics Committee of the Air Force Military Medical University. Mice (n = 6 per group) were sacrificed by intraperitoneal injection with sodium pentobarbital (50 mg/kg) at 16 weeks after HFD or NCD, and at 12 weeks after surgery.

#### 2.2.2 Sleeve gastrectomy

After 16 weeks of HFD, mice underwent SG or sham surgery, and the surgical approach was referred to in the previous research (39). In the sham group, only the abdomen was opened and sutured, but the same duration of anesthesia was ensured. 10% sugar water was given from 6 hours postoperatively and a liquid diet was started after 24 hours, and a normal HFD was started after 3 days. At the same time, the sugar water was replaced with normal drinking water.

#### 2.2.3 Glucose and lipid metabolism analysis

Intraperitoneal glucose tolerance test (IPGTT): After six hours of fasting, mice in each group were intraperitoneally injected with 20 percent glucose (1 g/kg). Tail vein glucose levels were then recorded using a glucometer (Roche, Germany) before, 15, 30, 60, 90, and 120 minutes after the injection. Intraperitoneal insulin tolerance test (IPITT): The mice in every group were fasted for six hours before receiving an intraperitoneal injection of insulin (0.05 U/kg). Blood glucose levels were taken in the tail vein before the injection and 15, 30, 60, 90, and 120 minutes later. The free fatty acid (FFA) in serum samples was tested with the FFA Content Assay Kit (BC0590, Solarbio, Beijing, China).

#### 2.2.4 Histological analysis

Liver specimens were fixed in 4% formalin buffer, paraffin-embedded, and serial 4 μm thick sections were used for HE staining to evaluate hepatocyte morphology. To assess hepatic steatosis, the frozen section oil red O staining of the fixed liver specimens was performed.

#### 2.2.5 Quantitative PCR analysis

TRIzol reagent (DP419, Tiangen, Beijing, China) was used to extract total RNA, and then employing a PrimeScript RT reagent Kit (RR037A, Takara, Dalian, China) for reverse transcription into cDNA. The PCR-amplification products were quantified using TB Green (RR820A, Takara, Dalian, China). As directed by the manufacturer, qPCR assays were run on the CFX Connect real-time PCR detection system (1855201, Biorad, USA). The associated genes’ mRNA expression levels were adjusted to match the level of the housekeeping gene β-actin. Primer information is in **Supplementary Table 1**.

#### 2.2.6 Statistical analysis

All the bioinformatics studies in this research were statistically analyzed using R software (V.4.1.2), and the rest were statistically analyzed using GraphPad Prism V.8 (GraphPad Software, La Jolla, California, USA). The significant differences between the groups were analyzed with Student’s t-test (parametric samples) and Wilcoxon signed-rank test (non-parametric samples). A p value of < 0.05 was considered statistically significant. Significance was represented by *p < 0.05, **p < 0.01 and ***p < 0.001. Error bars used the Standard error of mean (SEM).

## 3 Results

### 3.1 Identification of DEGs between NASH patients and healthy individuals

**Figure 1:**
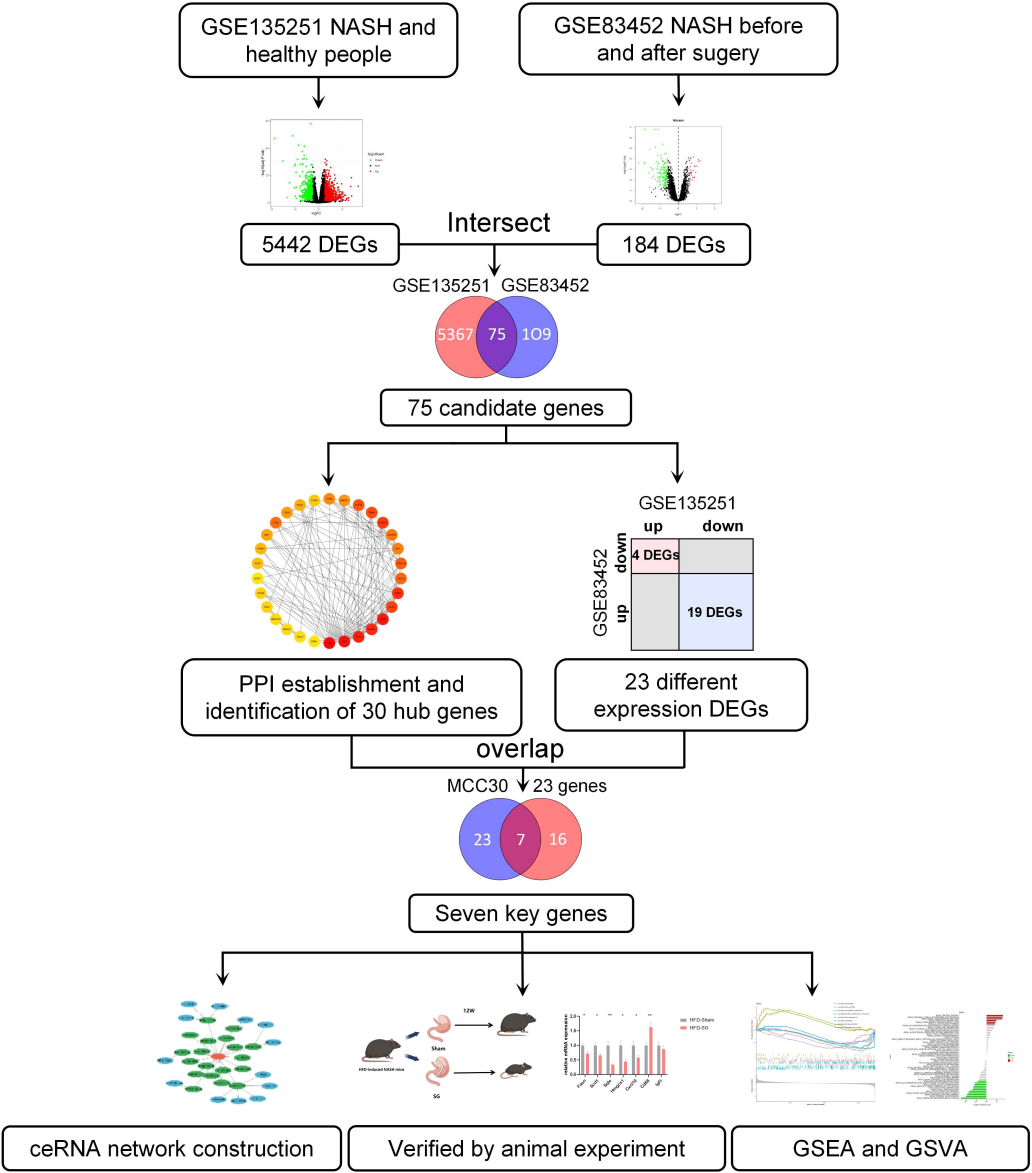
The work flowchart in this study.

**Figure 2:**
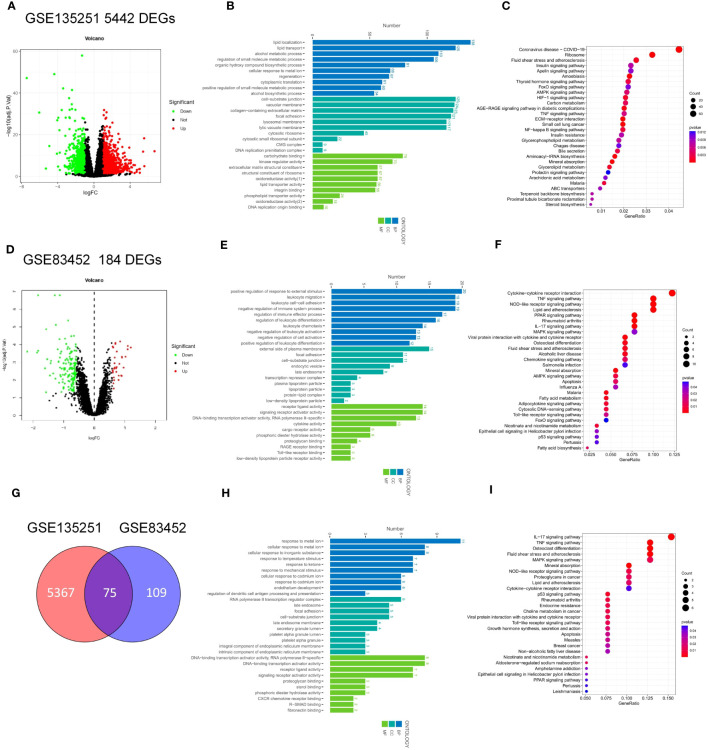
Identification and Enrichment analysis of DEGs in GSE135251 and GSE83452. **(A)** Volcano plots of gene expression in GSE135251, with the threshold of P<0.05 and |log FC| > 0.5. **(B)** GO enrichment analysis of the DEGs in GSE135251. **(C)** KEGG enrichment analysis of the DEGs in GSE135251. **(D)** Volcano plots of gene expression in GSE83452. **(E)** GO enrichment analysis of the DEGs in GSE83452. **(F)** KEGG enrichment analysis of the DEGs in GSE83452. **(E)** Venn diagram of the common DEGs in GSE135251 and GSE83452. **(F)** GO enrichment analysis of the common DEGs. **(G)** KEGG enrichment analysis of the common DEGs.

The workflow of this article was illustrated in **Figure 1**. To investigate the DEGs associated with NASH, we compared the transcriptome information of 155 NASH patients and 10 healthy individuals in GSE135251. A total of 5442 DEGs were obtained, 2944 DEGs were upregulated and 2498 DEGs were downregulated (**Figure 2A**). Gene ontology (GO) analysis and KEGG enrichment analysis were performed to elucidate the biological pathways associated with DEGs. Detailed information about the GO analysis was shown in **Figure 2B**. DEGs were mainly enriched in lipid localization and lipid transport in biological process (BP). They were associated with the cellular component (CC) such as the cell-substrate junction, vacuolar membrane and lysosomal membrane. Pathways related to molecular function (MF) include carbohydrate blinding, kinase regulator activity, oxidoreductase activity, and lipid transporter activity. The analysis of KEGG signaling pathways revealed that DEGs were primarily linked to the insulin, FoxO, apelin, and AMPK signaling pathways, which are involved in signaling pathways that regulate inflammation and glycolipid metabolism (**Figure 2C**).

### 3.2 Identification of DEGs before and after bariatric surgery

To investigate the key factors for remission of NSAH by bariatric surgery, we chose the GSE83452 dataset and selected 14 patients to compare baseline (NASH liver specimens obtained during bariatric surgery) and follow-up (NASH remission at one year after bariatric surgery) transcriptomic data. A total of 184 DEGs were identified compared to the baseline, with 32 genes upregulated and 152 genes downregulated after bariatric surgery (**Figure 2D**). The BP of the GO dataset was mainly enriched in leukocyte migration and leukocyte cell-cell adhesion. CC was most significantly enriched in the external side of plasma membrane, focal adhesion, and enriched in plasma lipoprotein particle, lipoprotein particle, and protein-lipid complex, which are closely related to lipid metabolism. DEGs were also enriched in MF with cytokine activity, RAGE receptor binding, and cargo receptor activity (**Figure 2E**). The three most frequently enriched pathways for DEGs in the KEGG database were the TNF signaling pathway, PPAR signaling pathway, and Rheumatoid arthritis (**Figure 2F**). The remaining signaling pathways were mainly associated with inflammation, lipid metabolism, and immunity.

### 3.3 Identification of common DEGs between GSE135251 and GSE83452

The DEGs in GSE135251 and GSE83452 were intersected using the Venn diagram, and a total of 75 common genes were obtained (**Figure 2G**). To understand the functions of these common DEGs, GO and KEGG enrichment analyses were performed. In GO analysis, it was found that BP was mainly focused on the response to metal ions, such as cellular response to cadmium ion, response to metal ion, etc. Pathways related to CC were mainly enriched in RNA polymerase II transcription regulator complex and platelet alpha granule lumen. The most significant differences in MF statistics were in proteoglycan binding, DNA-binding transcription activator activity, and RNA polymerase II-specific (**Figure 2H**). The remaining pathways were mainly related to inflammation and chemokines. KEGG analysis showed that the candidate genes were mainly enriched in pathways related to inflammation such as the IL-17 signaling pathway, TNF signaling pathway, and p53 signaling pathway. Other pathways Lipid and atherosclerosis, non-alcoholic fatty liver disease, and PPAR signaling pathway are related to lipid metabolism. The rest are Mineral absorption, Osteoclast differentiation, Rheumatoid arthritis, etc (**Figure 2I**).

### 3.4 PPI network construction and key genes identification

**Figure 3:**
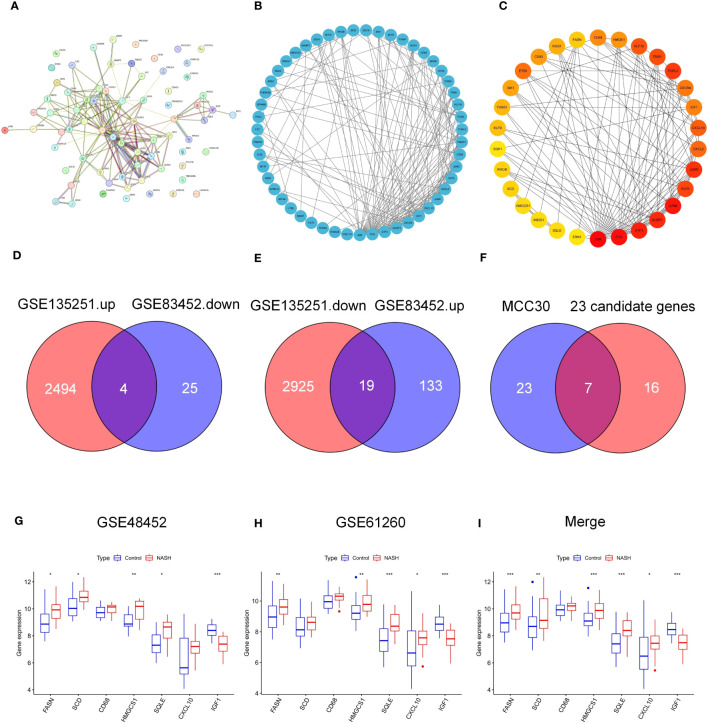
PPI network of common genes and identification of key genes. **(A–C)** PPI nrtwork of 75 common DEGs in GSE135251 and GSE83452. **(D)** Venn diagram of candidate DEGS down-regulation in GSE135251 and up-regulation in GSE83452. **(E)** Venn diagram of candidate genes up-regulation in GSE135251 and down-regulation in GSE83452. **(F)** Venn diagram of top 30 DEGs of PPI and 23 candidate genes in **(D, E)**, obtained 7 key genes (FASN, SCD, CD68, HMGCS1, SQLE, CXCL10, IGF-1). **(G)** 7 key genes expression in NASH dataset GSE48452. **(H)** 7 key genes expression in NASH dataset GSE61260. **(I)** Merge the expression of 7 key genes in two datasets **(G, H)**.

To determine the interaction of 75 common genes and screen hub genes, we constructed a PPI network of 75 common genes using the STRING online database (**Figure 3A**). Next, we hid separate nodes in the network, 51 genes remained in the network and were visualized by Cytoscape (**Figure 3B**). Subsequently, the top 30 hub genes for the MCC algorithm were identified by the CytoHubba plugin (**Figure 3C**). To identify the key factors of bariatric surgery to alleviate NASH, we intersected the DEGs downregulated in GSE135251 with those upregulated in GSE83452, to obtain a total of 4 candidate genes (**Figure 3D**). The DEGs upregulated in GSE135251 and downregulated in GSE83452 were overlapped to obtain 19 candidate genes (**Figure 3E**). The top 30 genes of PPI were considered to intersect with 23 candidate genes, and finally, seven key genes were obtained (FASN, HMGCS1. SQLE, SCD1, CXCL10, CD68, IGF1, **Figure 3F**). After bariatric surgery, the expressions of FASN, HMGCS1.SQLE, SCD1, and CXCL10 were decreased, while the expression of IGF1 was increased (**Figure 1**). To verify the accuracy of the selection of these seven key genes, we obtained similar results in the NASH-related datasets GSE48452 (**Figure 3G**) and GSE61260 (**Figure 3H**). Merging the results from the two datasets yielded similar results (**Figure 3I**). FASN, HMGCS1.SQLE, SCD1, CXCL10, and IGF1 showed the same trend and were statistically significant. Although there was no statistical difference in CD68, the trend was the same.

### 3.5 Functional enrichment of key genes

**Figure 4:**
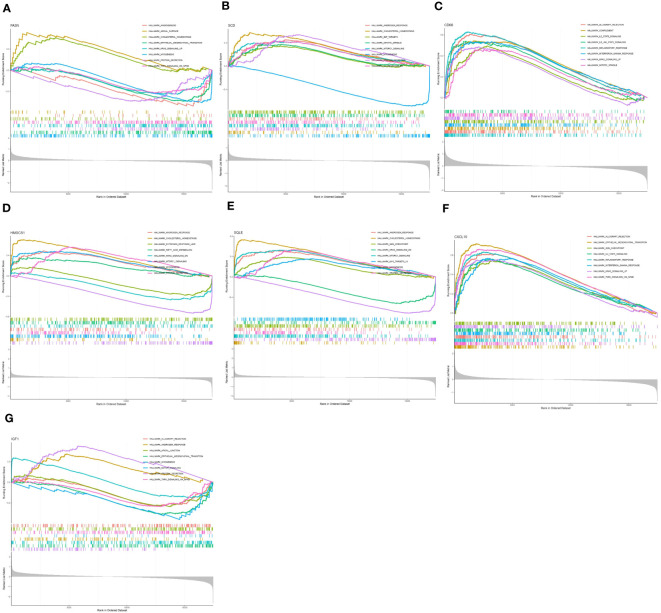
Gene set enrichment analysis (GSEA) of key genes in HALLMARK gene set. GSEA of FASN **(A)**, SCD **(B)**, CD68 **(C)**, HMGCS1 **(D)**, SQLE **(E)**, CXCL10 **(F)**, and IGF1 **(G)** in HALLMARK gene set.

To further study the potential role of seven key genes in improving NASH after bariatric surgery, we performed GSEA and GSVA analysis. In the HALLMARK gene set, the GSEA results showed that the ‘cholesterol homeostasis’ pathway was enriched in the FASN, SCD1, HMGCS1, and SQLE high expression groups (**Figures 4A, B, D, E**), and the ‘protein section’ pathway was enriched in the SCD, HMGCS1, SQLE, and IGF1 high expression group, but in the FASN low expression group (**Figures 4A, B, D, E, G**). The ‘myogenesis’ pathway was enriched in the FASN, SCD1, HMGCS1, and SQLE low expression groups (**Figures 4A, B, D, E**). The ‘allograft rejection’, ‘IL2 STAT5 signaling’, ‘inflammatory response’, ‘interferon gamma response’, and ‘KRAS signaling up’ pathways were enriched in the CD68 and CXCL10 high expression groups (**Figures 4C, F**). In the KEGG gene set, the GSEA results are shown in **Supplementary Figure 1**.

**Figure 5:**
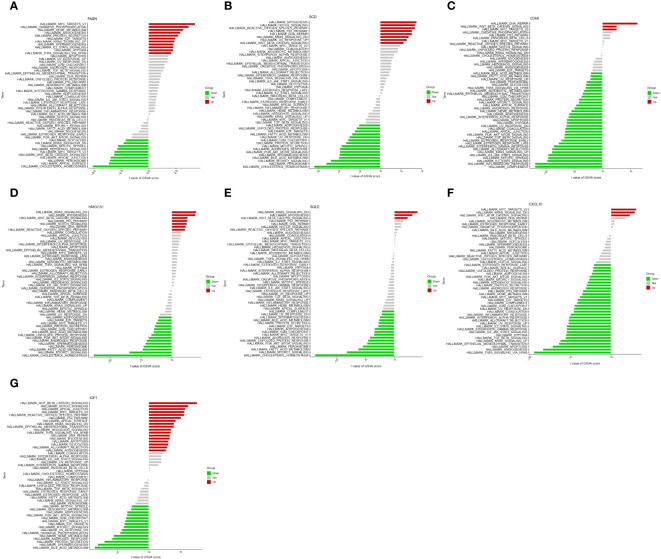
Gene set variation analysis (GSVA) of key genes in HALLMARK gene set. GSVA of FASN **(A)**, SCD **(B)**, CD68 **(C)**, HMGCS1 **(D)**, SQLE **(E)**, CXCL10 **(F)**, and IGF1 **(G)** in HALLMARK gene set.

The GSVA results of the HALLMARK gene set are shown in **Figure 5**, ‘KRAS signaling DN’ pathway was enriched in the FASN, SCD, HMGCS1, SQLE, CXCL10, and IGF1 high expression groups (**Figures 5A, B, D–G**) and CD68 low expression groups (**Figure 5C**). ‘WNT β catenin signaling’ pathway was enriched in the SCD, CD68, HMGCS1, SQLE, CXCL10, and IGF1 high expression groups (**Figures 5B, C, D–G**) and FASN low expression groups (**Figure 5A**). Different from GSEA, the ‘cholesterol homeostasis’ pathway was enriched in the FASN, SCD, HMGCS1, and SQLE low expression groups (**Figures 5A, B, D, E**). The results in the KEGG gene set are shown in **Supplementary Figure 2**.

### 3.6 ceRNA network construction of key genes

**Figure 6:**
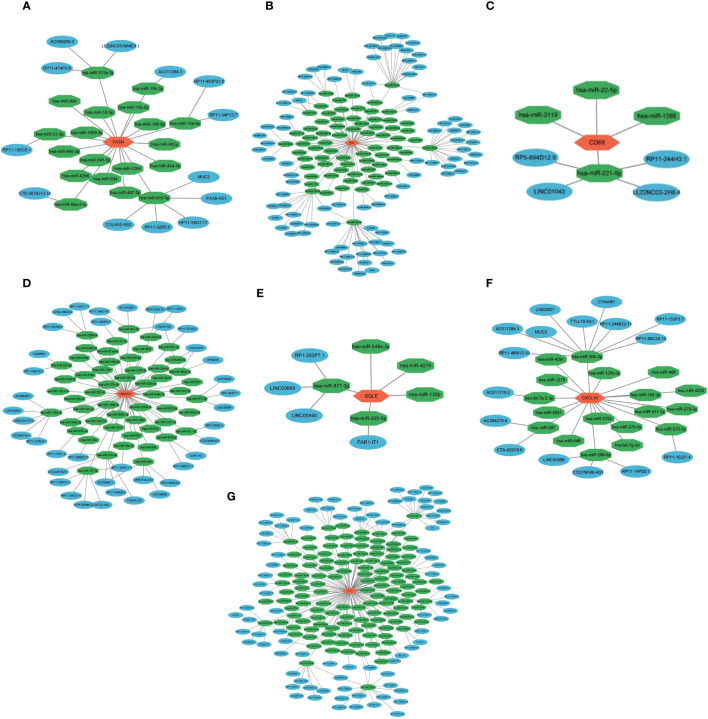
ceRNA network construction of key genes. ceRNA network construction of FASN **(A)**, SCD **(B)**, CD68 **(C)**, HMGCS1 **(D)**, SQLE **(E)**, CXCL10 **(F)**, and IGF1 **(G)**.

**Figure 7:**
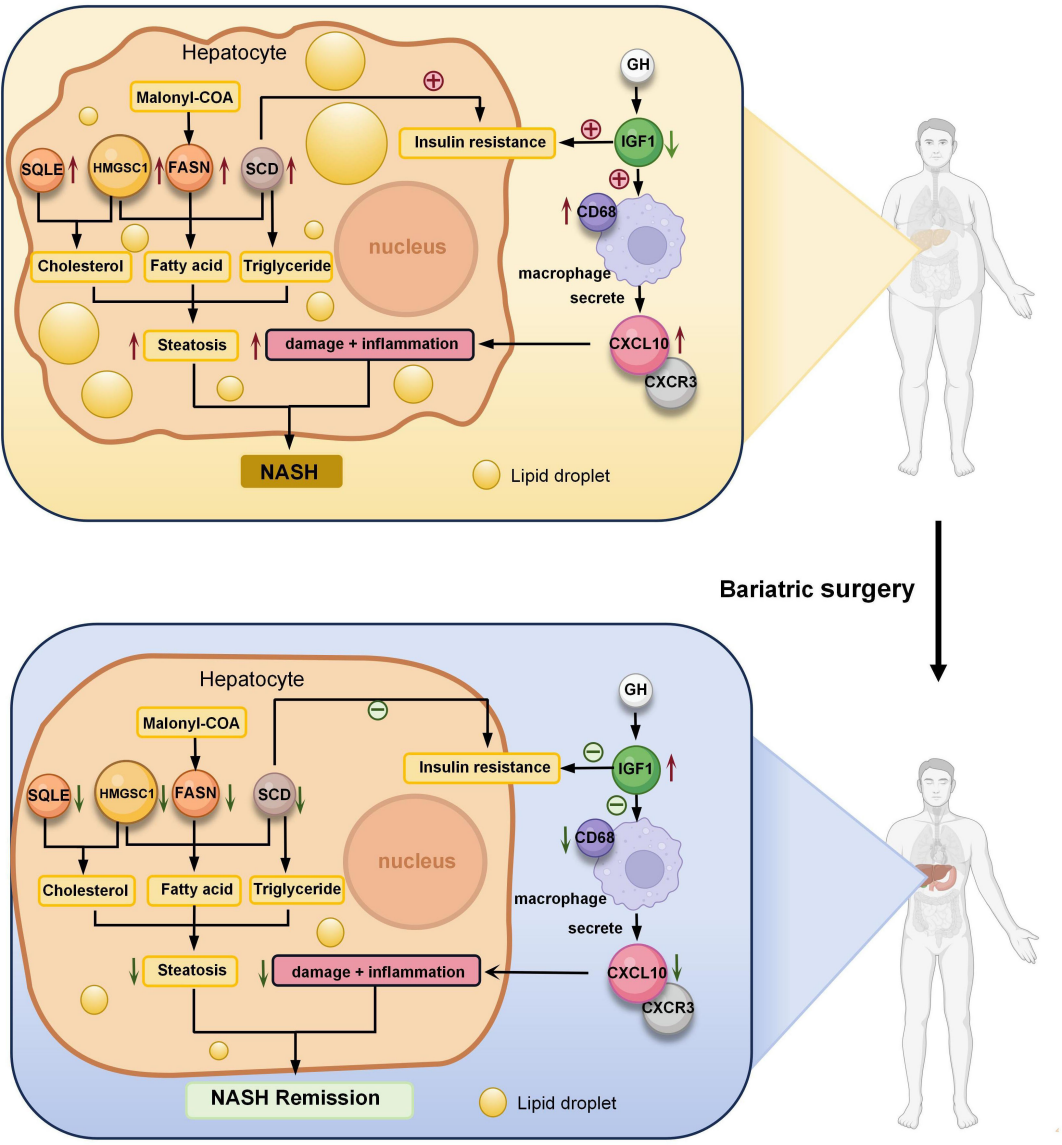
Role of seven key genes in remission of NASH after bariatric surgery. Fatty acid synthase (FASN) is a key enzyme in fatty acid synthesis. Malonyl-CoA is changed into fatty acids (FA) by FASN. Steroidal coenzyme A desaturase (SCD) is the rate-limiting enzyme of monounsaturated fatty acids and a key enzyme in the synthesis of triglycerides (TG). It has been observed that lowering SCD enhances insulin sensitivity. Hydroxy-3-methylglutaryl-CoA synthetase 1 (HMGCS1) is a protein-coding gene associated with cholesterol (TC) biosynthesis and steroid metabolism and promotes FA synthesis and disruption of lipid metabolism. The enzyme squalene epoxidase (SQLE) limits the rate at which TC is synthesized and encourages the build-up of TC and cholesteryl esters in hepatocytes. C-X-C motif chemokine 10 (CXCL10) is secreted by macrophages and causes an inflammatory cascade response when it interacts with its cognate receptor C-X-C motif receptor 3 (CXCR3), resulting in inflammation and hepatocyte damage. the expression of hepatic inflammation-related marker CD68 was elevated in NASH. Insulin-like growth factor 1 (IGF1) is primarily produced by growth hormone (GH) stimulated hepatic production in adults. IGF1-induced insulin sensitization has been in rodent models of liver disease, including models of NAFLD and NASH. And IGF1 can have an anti-inflammatory effect by acting on macrophages. Bariatric surgery alleviates NASH by altering the expression of these seven key genes, reducing liver steatosis, hepatocyte damage, inflammation.

We created a ceRNA network construction to investigate what influences the regulation of key genes. By binding to mRNAs, microRNAs could silence genes, but ceRNAs-which include circRNAs and lncRNAs-can control gene expression by competitively binding to microRNAs (40). A ceRNA can bind multiple microRNAs, and the microRNA binding sites on ceRNAs are called microRNA recognition elements (MREs) (41). Normally there are one or more MREs on the ceRNAs, and as the expression of the ceRNAs increases, the microRNAs compete for binding, leading to an increase in the transcription level of the mRNAs and ultimately an increase in the expression level of the proteins and vice versa (41, 42). We constructed a ceRNA network of seven key genes (**Figures 6A–G**), indicating the miRNAs that can bind to the mRNAs of the key genes and the ceRNAs that can bind to the miRNAs. We established the role of seven key genes in NASH remission after bariatric surgery (**Figure 7**).

### 3.7 The expression level of key genes and glycolipid metabolism in NASH mouse models

**Figure 8:**
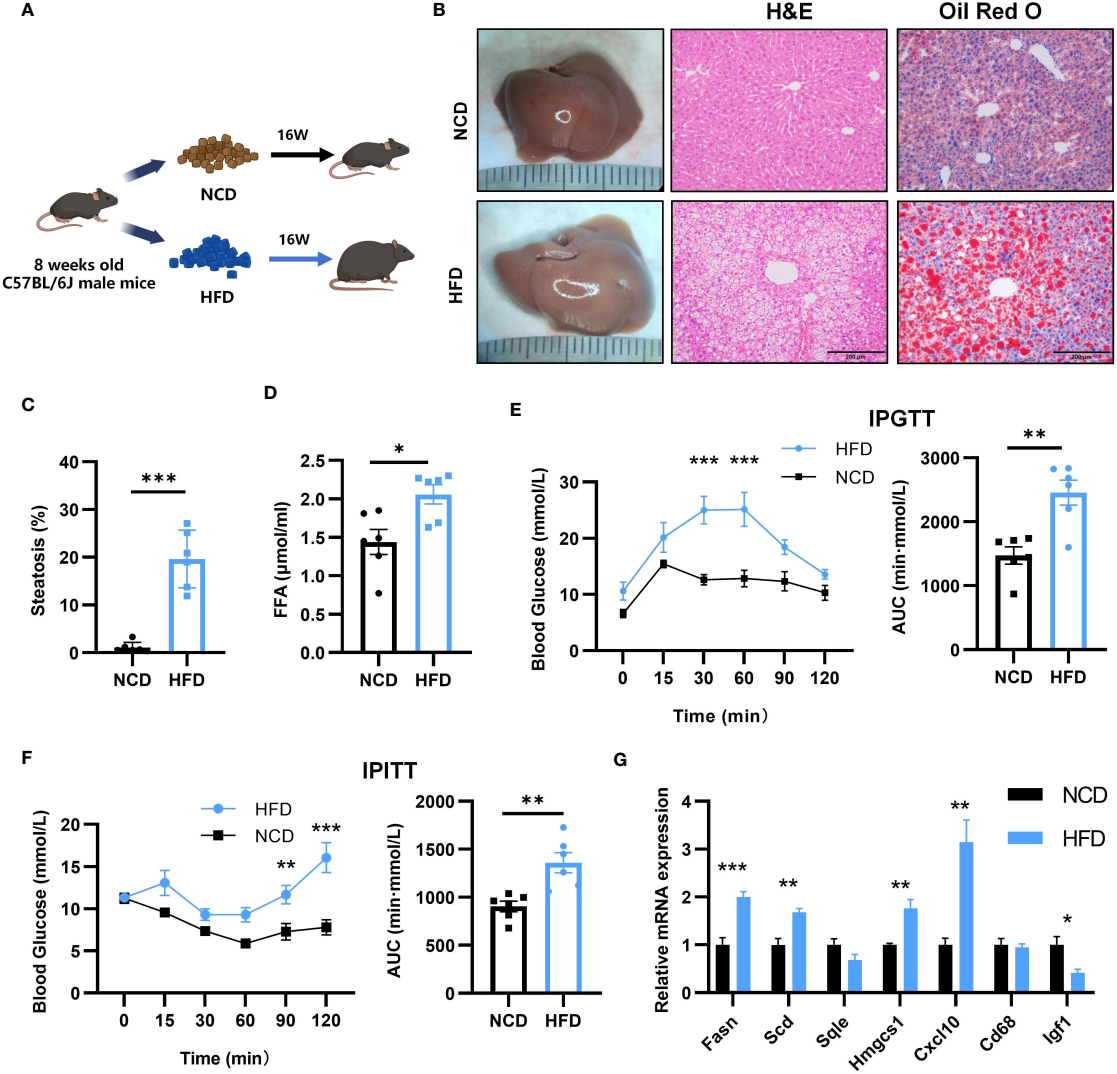
Establishment of mouse NASH model and key gene expression. **(A)** Flow chart of HFD-NASH mice (n=6/group). **(B)** Gross morphology, H&E staining and Oil Red O staining of hepatic tissue from control and HFD diet mice (16 weeks on the indicated diet, n=6/group), Scale bar: 200mm. **(C)** Percentage of liver tissue with steatosis. **(D)** Intraperitoneal glucose tolerance test (IPGTT) and the areas under the curve (AUC) at 16 weeks after the indicated diet (n=6/group). **(E)** Serum free fatty acid (FFA) content at 16 weeks after indicated diet (n=6/group). **(F)** Intraperitoneal insulin tolerance test (IPITT) and AUC at 16 weeks after indicated diet (n=6/group). **(G)** Relative mRNA expression in the liver of 7 key genes by qPCR (16 weeks after indicated diet, n=6/group). All Figures: ns, no significance; *p < 0.05, **p < 0.01, ***p < 0.001.

To verify the expression of key genes and glycolipid metabolism in NASH, we induced a mouse NASH model with HFD (**Figure 8A**). After 16 weeks of HFD, the liver of the mice increased in size and yellow granules were visible to the naked eye (**Figure 8B**). HE staining and oil red O staining were performed on the livers (**Figure 8B**), which showed balloon-like hepatocytes, inflammatory cell infiltration, and a large number of lipid droplet aggregates in the livers of HFD mice. The percentage of steatosis of liver tissue is shown in the **Figure 8C**. Meanwhile, free fatty acids (FFA) in serum were significantly elevated (**Figure 8D**). IPGTT found that blood glucose in the HFD group decreased slowly after increasing blood glucose, with an increase in the area under the curve (AUC) and impaired glucose tolerance (**Figure 8E**). IPITT found that after insulin injection, blood glucose decreased more slowly in the mice of the HFD group than that of the NCD group, the AUC was higher than that of the NCD group, and insulin sensitivity was reduced (**Figure 8F**). We verified the expression of seven key genes in NASH mice by qPCR. Compared with NCD, HFD mice showed upregulated expression of FASN, SCD1, HMGCS1 and CXCL10 and reduced expression of IGF1, which was consistent with the results of bioinformatics analysis. However, no significant changes were detected in the expression of SQLE and CD68 (**Figure 8G**).

### 3.8 The expression level of key genes and glycolipid metabolism in NASH mouse models after SG

**Figure 9:**
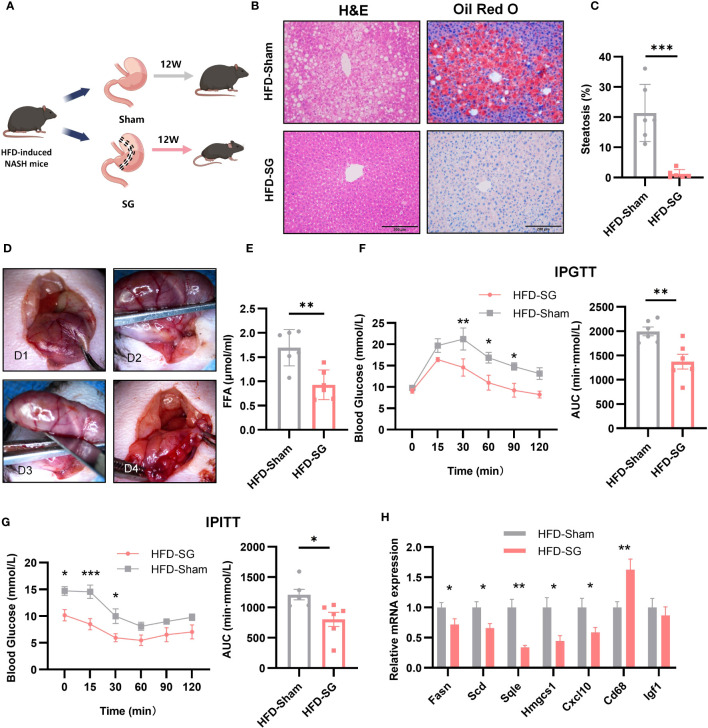
The expression of 7 key genes after SG. **(A)** Flow chart of SG (n=6/group). **(B)** HE staining and Oil Red O staining of liver tissue from sham and SG group (8 weeks post-surgery, n=6/group), Scale bar: 200mm. **(C)** Percentage of liver tissue with steatosis. **(D)** The main procedure of SG; a: Expose the body of stomach; b: Fix the gastric body with ophthalmic forceps; c: Excise the greater curvature of stomach; d: Suture stump. **(E)** IPGTT and AUC at 8 weeks post-surgery (n=6/group). **(F)** Serum FFA content at 8 weeks post-surgery (n=6/group). **(G)** IPITT and AUC at 8 weeks post-surgery (n=6/group). (H) Relative mRNA expression in the liver of 7 key genes by qPCR at 8 weeks post-surgery (n=6/group).

To explore the effect of bariatric surgery on the expression of key genes and metabolic changes in NASH mice, we performed SG on NASH mice. 12 weeks after SG (**Figures 9A, D**), the liver of mice had a regular liver arrangement, ballooning hepatocytes, inflammatory cells and fat granules were significantly reduced compared to the Sham group (**Figure 9B**). The percentage of steatosis of liver tissue is shown in the **Figure 9C**. Serum FFA was significantly decreased (**Figure 9E**). The IPTGG results showed that blood glucose increased slowly and decreased in magnitude in the SG group, with a lower AUC and an increase in glucose tolerance (**Figure 9F**). IPITT results showed that the curve decreased in the SG group, the AUC decreased, and insulin sensitivity increased (**Figure 9G**). The expression of seven key genes in SG mice was verified by qPCR (**Figure 9H**). Compared with the Sham group, the expression of FASN, SCD, HMGCS1, CXCL10, and SQLE was downregulated, and the expression of CD68 was upregulated, but the expression of IGF1 did not show any significant change. Among them, FASN, SCD1, HMGCS1, CXCL10, and SQLE expressions were consistent with the analyzed results. Combined with the qPCR results of NASH mice, all four genes of FASN, SCD1, HMGCS1, and CXCL10 were consistent with the biological analysis.

## 4 Discussion

With the rising prevalence of obesity, NAFLD has become the most prevalent chronic liver disease worldwide (43). Despite many advances in disease research, there are still no FDA-approved medications for NASH (44). Current evidence suggests that weight reduction is an effective way to alleviate NAFLD (45). However, achieving sustained and significant weight loss through dietary improvements and increased exercise remains challenging. Bariatric surgery is effective in reducing weight and alleviating metabolism-related diseases such as T2DM and dyslipidemia, making it a promising long-term treatment option for NASH (46). Although it is uncontroversial that bariatric surgery relieves NASH, the underlying mechanisms remain unclear. Bioinformatic methods have been widely used to mine transcriptomic information to explore the key factors affecting disease onset and progression. Therefore, we performed a whole transcriptome analysis using bioinformatic methods to search for key genes that may mediate bariatric surgery to alleviate NASH. Additionally, we established a mouse model of NASH and performed qPCR assays to verify the expression of key genes.

In this study, we included two microarray studies comparing transcriptome information from NASH patients and healthy individuals in GSE135251. A total of 5442 DEGs were obtained, 2944 DEGs were upregulated and 2498 DEGs were downregulated (**Figure 2A**). Also, when comparing baseline (NASH liver samples collected during bariatric surgery) and follow-up (patients with NASH remission one year after bariatric surgery) in GSE83452, a total of 184 DEGs were obtained, with 32 genes upregulated and 152 genes downregulated in the postoperative DEGs (**Figure 2D**). Intersecting the two datasets yielded a total of 75 candidate genes (**Figure 2G**). To identify the key factors for the alleviation of NASH by bariatric surgery, we intersected the genes with different expressions in the two datasets and obtained a total of 23 candidate genes (**Figures 3D, E**). Using the PPI network, the top 30 DEGs were sorted by degree value and taken as candidate genes. Seven key genes (FASN, HMGCS1. SQLE, SCD1, CXCL10, CD68, IGF1, **Figure 3F**) were finally obtained by intersecting with 23 candidate genes. Among them, FASN, HMGCS1, SQLE, SCD1, CXCL10, and CD68 were downregulated and IGF1 was upregulated after bariatric surgery.

Fatty acid synthase (FASN) is a pivotal enzyme in the process of fatty acid synthesis. The initial step in hepatic lipogenesis involves the conversion of citrate to acetyl-coenzyme A (CoA) by ATP citrate lyase. Acetyl-CoA Carboxylases (ACC) 1 and 2 next convert CoA to malonyl-CoA, which is then transformed into fatty acids by FASN (47). FASN expression is increased in NAFLD patients and HFD mice (48, 49), and excess fatty acids in the animals can form fats through esterification, which increases fat deposition in the animals. However, studies on FASN expression after bariatric surgery are lacking. In this experiment, we found that the expression of FASN was reduced after SG. This reduction suggests that bariatric surgery may reduce fatty acid synthesis by down-regulating FASN expression, thereby reducing the deposition of lipids in the liver and alleviating NAFLD. At the same time, the reduction in FASN expression leads to the accumulation of malonyl-CoA, which acts on the hypothalamus to suppress appetite and induce significant weight loss and fat reduction (50).

Steroidal coenzyme A desaturase (SCD) is the rate-limiting enzyme of monounsaturated fatty acids and a key enzyme in the synthesis of triglycerides (TG) (51). SCD also known as SCD1, was estimated to be higher in NASH patients than in patients with normal liver function (52), while SCD expression was increased in HFD-induced NASH mice (**Figure 8G**), suggesting that high SCD activity is associated with NASH. Hepatic SCD expression is required for carbohydrate-induced obesity (53). In rodents, downregulation of SCD expression reduces body fat, increases energy expenditure, and upregulates the expression of several genes for fatty acid β-oxidases in the liver. It has been observed that lowering SCD enhances insulin sensitivity and activates adenosine monophosphate-activated protein kinase (AMPK) (54). Intraperitoneal injection of SCD-targeted antisense oligonucleotide (ASO) in mice inhibits SCD in the liver and adipose, resulting in increased insulin sensitivity, decreased hepatic fatty acid synthesis, and prevention of HFD-induced obesity and hepatic steatosis (55). Lipid metabolism plays a key role in the onset of insulin resistance and diabetes, and insulin resistance is a direct result of obesity and the buildup of extra lipids in non-adipose tissues. Therefore, increased insulin sensitivity in SCD-deficient mice is predicted by the reduced lipid synthesis and increased lipid oxidation seen in these animals. In hepatic stellate cells, reduced SCD expression resulted in a reversal of their fibrotic phenotype (56). Aramchol is an SCD inhibitor in a Phase II B clinical study of the effect of Aramchol on NASH. Compared to the placebo group, the Aramchol group had significantly lower liver fat content (p = 0.045) and a higher rate of NASH remission (16.7% vs 5%, OR = 4.74; p = .0514) (57). However, the effect of bariatric surgery on SCD expression is missing. In this study, SG reduced SCD expression (**Figure 9H**), one of the important factors in the theory of palliation of NASH by bariatric surgery.

Hydroxy-3-methylglutaryl-CoA synthetase 1 (HMGCS1) is a protein-coding gene associated with cholesterol biosynthesis and steroid metabolism (58). HMGCS1 expression is upregulated in NASH mice (**Figure 8G**), which promotes fatty acid synthesis and disruption of lipid metabolism (59) and facilitates intrahepatic lipid deposition, leading to excessive lipid content in the liver (60). Like regulating FASN, SCD1, SG also downregulated HMGCS1 expression. Rather than alleviating NASH by specifically reducing the expression of a particular gene, SG seems to restore insulin sensitivity (**Figure 9G**), inhibit fatty acid synthesis (**Figure 9E**), and reduce lipid deposition in the liver by attenuating the expression of FASN, SCD1, and HMGCS1 (**Figure 9H**).

The enzyme squalene epoxidase (SQLE) limits the rate at which cholesterol is synthesized and encourages the build-up of cholesterol and cholesteryl esters in hepatocytes (61). In both NASH mouse models and humans, SQLE is expressed at a high level. However, in this experiment, there was no significant difference in SQLE expression in NASH mice (**Figure 8G**). Meanwhile, in a porcine NASH model, SQLE was negatively correlated with hepatic lipid droplet area (62). The reason for this may be due to insufficient modeling time and species differences. SQLE overexpression in transgenic TG mice leads to hepatic cholesterol accumulation, which triggers pro-inflammatory nuclear factor-kB signaling and steatohepatitis. By directly binding to carbonic anhydrase III (CA3), SQLE triggers the activation of sterol regulatory element binding protein 1C, as well as the expression of SCD, FASN, and acetyl coenzyme A carboxylase, which in turn triggers *de novo* hepatic lipogenesis (63). Through the induction of cholesterol biosynthesis and adipogenesis, which is mediated by the SQLE/CA3 axis, SQLE drives the initiation and progression of NASH. It has been demonstrated that targeting SQLE and CA3 together is effective in treating NASH (63). Decrease in SQLE expression after SG surgery (**Figure 9H**), thereby reducing cholesterol synthesis. Sharing a similar function with FASN, SCD, and HMGCS1, i.e., being involved in explaining the reduction of hepatic fat deposition.

Persistent inflammation is an important factor in the progression of NASH. Chemokines, which control the movement and activity of hepatocytes, Kupffer cells, hepatic stellate cells, endothelial cells, and circulating immune cells, regulate hepatic inflammation (64). A chemotactic ligand known as C-X-C motif chemokine 10 (CXCL10), which is secreted by macrophages, causes an inflammatory cascade response when it interacts with its cognate receptor C-X-C motif receptor 3 (CXCR3). CXCL10, highly expressed in NASH mice (**Figure 8G**), targets CXCR3 to directly cause hepatocyte damage, resulting in inflammation and liver injury (65). This may mediate macrophage-associated inflammation in NASH mouse models. Macrophage p38α induces the secretion of pro-inflammatory cytokines such as CXCL10, IL6, and TNFα, leading to M1 macrophage polarization and exacerbation of steatohepatitis alterations in hepatocytes (66). In CXCL10 knockout mice, hepatic inflammation, subsequent hepatic injury, and fibrosis were reduced (67). SG likely attenuates hepatocyte damage and liver tissue inflammation by reducing CXCL10 expression in mice (**Figure 9H**).

CD68 is used as a marker for macrophages, and its high expression is closely associated with hepatic inflammation (68). In the present study, different from the results from bioinformatics analysis, we have noticed that qPCR results have shown that there was an increase in CD68 expression in livers of NASH mice, when compared to that of Sham group (**Figure 9H**). There are two reasons to explain the difference. First, the objects of bioinformatics analysis and qPCR are so different. In the bioinformatics analysis, all databases are from patients, while qPCR results are obtained from experimental mice. Second, the time points of bioinformatics analysis and qPCR are quite different. The time point on patients for the comparison of CD68 expression levels in bioinformatics analysis is 52 weeks after bariatric surgery (SG or RYGB), while the time point on mice for the comparison of CD68 expression levels by qPCR is 12 weeks after SG surgery. It is deduced that NASH patients receiving surgery after more than one year have improved so greatly that CD68 expression might return to the normal level. Different from the long recovery course of patients, the livers of NASH mice after 12 weeks after SG operations have been still suffered from inflammation attack, therefore it is reasonable for the absence of the decrease of CD68 expression. Our present result is consistent with previous study which has reported the increased expression of CD68 in adipose tissue of mice after SG (69). It is deduced that the increase of CD68 level could represent the presence of inflammatory state.

Insulin-like growth factor 1 (IGF1) is primarily produced by growth hormone (GH) stimulated hepatic production in adults (70). GH primarily affects metabolism by stimulating lipolysis in white adipose tissue, raising free fatty acid levels in the blood, blocking glucose oxidation, and decreasing insulin sensitivity in the liver and peripheral tissues (71). An increasing body of research indicates that IGF1 directly targets the liver, as well as hepatocytes, macrophages, and hematopoietic stem cells, through a variety of mechanisms that inhibit the progression of nonalcoholic fatty liver disease (72). It was verified in this experiment that IGF1 expression levels were reduced in NASH mice (**Figure 8G**). GH and IGF1 have also been reported to reduce oxidative stress in hepatocytes, suggesting that they have different effects on the multiple strikes of NASH (73). In diet-induced obese mice, the knockdown of IGF1 receptors in macrophage precursor cells led to an increase in M1 macrophages and induced metabolic dysfunction, suggesting a protective effect against inflammation in the IGF1 signaling pathway in macrophages (74). IGF1-induced insulin sensitization has been in rodent models of liver disease, including models of NAFLD and NASH, and has been demonstrated to have antifibrotic properties (73, 75). However, no significant changes in IGF1 were seen after SG, suggesting that the recovery of insulin sensitivity in animals is not dependent on IGF1 concentration.

In GSEA, FASN, SCD, HMGCS1, and SQLE high-expression groups are enriched in the ‘cholesterol homeostasis’ pathway. It is suggested that ‘cholesterol homeostasis’ is closely related to the development of NASH, and bariatric surgery may reduce lipid deposition by reducing the ‘cholesterol homeostasis’ pathway. The ‘allograft rejection’, ‘IL2 STAT5 signaling’, ‘inflammatory response’, ‘interferon gamma response’, and ‘KRAS signaling up’ pathways were enriched in the CD68 and CXCL10 high-expression groups (**Figures 4C, F**). It is suggested that the inflammatory pathway is closely related to the occurrence of NASH, and bariatric surgery may alleviate NASH by reducing the inflammation-related pathway.

NASH patients have increased *de novo* lipogenesis and increased fatty acid flux in the liver, leading to heightened production of lipotoxic substances that significantly contribute to hepatic inflammation and hepatocyte death associated with steatohepatitis (76). By down-regulating lipid metabolism-related genes (FASN, SCD, HMGCS), bariatric surgery reduces free fatty acid levels, restores insulin sensitivity, and reduces lipid deposition in the liver. Meanwhile, it may mitigate hepatocyte injury and liver tissue inflammation by down-regulating CXCL10 expression, thereby achieving the purpose of alleviating NASH. Recent data have revealed that myeloid cell-derived growth factor (MYDGF) alleviates NAFLD and inflammation in a manner involving IKKβ/NF-κB signaling, and serves as a factor involved in the crosstalk between the liver and bone marrow that regulates liver fat metabolism (77). Moreover, stem cell growth factor-beta (SCGF-β) has shown an activity on granulocyte/macrophage progenitor cells in combination with granulocyte macrophage colony-stimulating factor (GM-CSF) and macrophage colony-stimulating factor (M-CSF). In fact, in obesity patients with NAFLD, SCGF-β levels have been linked to insulin resistance and hepatic steatosis severity with the mediation role of CRP (78). In the present study, qPCR results have shown the increase of CD68 expression in livers of NASH mice receiving SG. We have discussed this might be due to the inflammatory infiltration of monocytes/macrophages into livers. This result is consistent with previous study that Da Riva et al. have reported that CD68-positivity could be detected in SCGF-positive areas, which has indicated the inflammatory infiltration state (79). It has been also noticed that Kim ML et al. have reported that the level of CXCL10 is elevated in sera from patients with acute rheumatic fever, and CXCL10 could bind to CXCR3 of T cells to activate the release of GM-CSF from T cells (80). This result is consistent with our present study that SG operation treatment could decrease the CXCL10 level of NASH mice. Therefore, growth factors, as key factors in NASH, may be potential therapeutic targets for NASH.

Bariatric surgery may be done by altering the expression of key genes (FASN, HMGCS1, SQLE, SCD, CXCL10, CD68, IGF1) to improve NASH. CeRNA network construction provides a new choice for regulating the expression of the key genes. In the future, the mRNA level of the target genes may be regulated by changing the level of related ceRNAs or microRNAs. The ultimate goal is to obtain remission of NASH without the use of a scalpel.

Limitations of this study

Although obvious changes in glucose and lipid metabolism were observed in HFD-NASH mice, the hepatic inflammation exhibited a mild phenotype. We verified that the key genes are limited to the mRNA level rather than the protein level, which does not reflect their final levels within the organism. Obesity, T2DM, sex and age may be the influential factors of NASH. However, this study did not account for potential confounding variables when screening DEGs. The ceRNA network construction only found microRNAs and ecRNAs that could regulate mRNA levels of key genes, but it was not verified in NASH mice and humans.

## 5 Conclusion

In this study, we obtained seven key genes (FASN, HMGCS1. SQLE, SCD1, CXCL10, CD68, IGF1) by analyzing transcriptomic information that may be the key to alleviating NASH after bariatric surgery. It was found that SG reduced FFA levels and lipid deposition in the liver by down-regulating lipid metabolism-related genes (FASN, SCD1, HMGCS1). Meanwhile, it may reduce hepatocyte injury and liver tissue inflammation by down-regulating CXCL10, to achieve the purpose of alleviating NASH. CeRNA network construction of the key genes provides a new choice for improving the NASH.

## Data availability statement

The datasets presented in this study can be found in online repositories. The names of the repository/repositories and accession number(s) can be found in the article/**Supplementary Material**.

## Ethics statement

The animal study was approved by Animal Welfare Ethics Committee of the Air Force Military Medical University. The study was conducted in accordance with the local legislation and institutional requirements.

## Author contributions

XC: Data curation, Formal analysis, Methodology, Validation, Writing – original draft. SD: Data curation, Formal analysis, Methodology, Software, Writing – original draft. YS: Data curation, Methodology, Writing – original draft. YW: Conceptualization, Project administration, Supervision, Writing – review & editing. YY: Conceptualization, Funding acquisition, Project administration, Supervision, Writing – review & editing. YB: Formal analysis, Methodology, Writing – original draft.
