# Supplementary material for: Key genes involved in nonalcoholic steatohepatitis improvement after bariatric surgery
Source: Front Endocrinol (Lausanne). 2024 Feb 26;15:1338889. doi: 10.3389/fendo.2024.1338889 (PMC10925704; doi:10.3389/fendo.2024.1338889)
Supplement: Supplementary file 1 [file DataSheet_1.docx]

Supplementary Material

Key genes involved in nonalcoholic

steatohepatitis improvement after bariatric surgery

Xiyu Chen^1^, Shi-Zhou Deng^1^, Yuze Sun^1^, Yunhu Bai^1,2^, Yayun Wang^3*^, Yanling Yang^1*^

^1^Department of Hepatobiliary Surgery, Xi-Jing Hospital, The Fourth Military Medical University, Xi’an, China.

^2^Department of General Surgery, 988 Hospital of Joint Logistic Support Force, Zheng Zhou, China.

^3^Specific Lab for Mitochondrial Plasticity Underlying Nervous System Diseases, National Demonstration Center for Experimental Preclinical Medicine Education, The Fourth Military Medical University, Xi’an, China.

*** Correspondence:**

Yanling Yang, Yayun Wang

[yangyanl@fmmu.edu.cn](mailto:yangyanl@fmmu.edu.cn), wangyy@fmmu.edu.cn

# Supplementary Figures and Tables

## Supplementary Figures


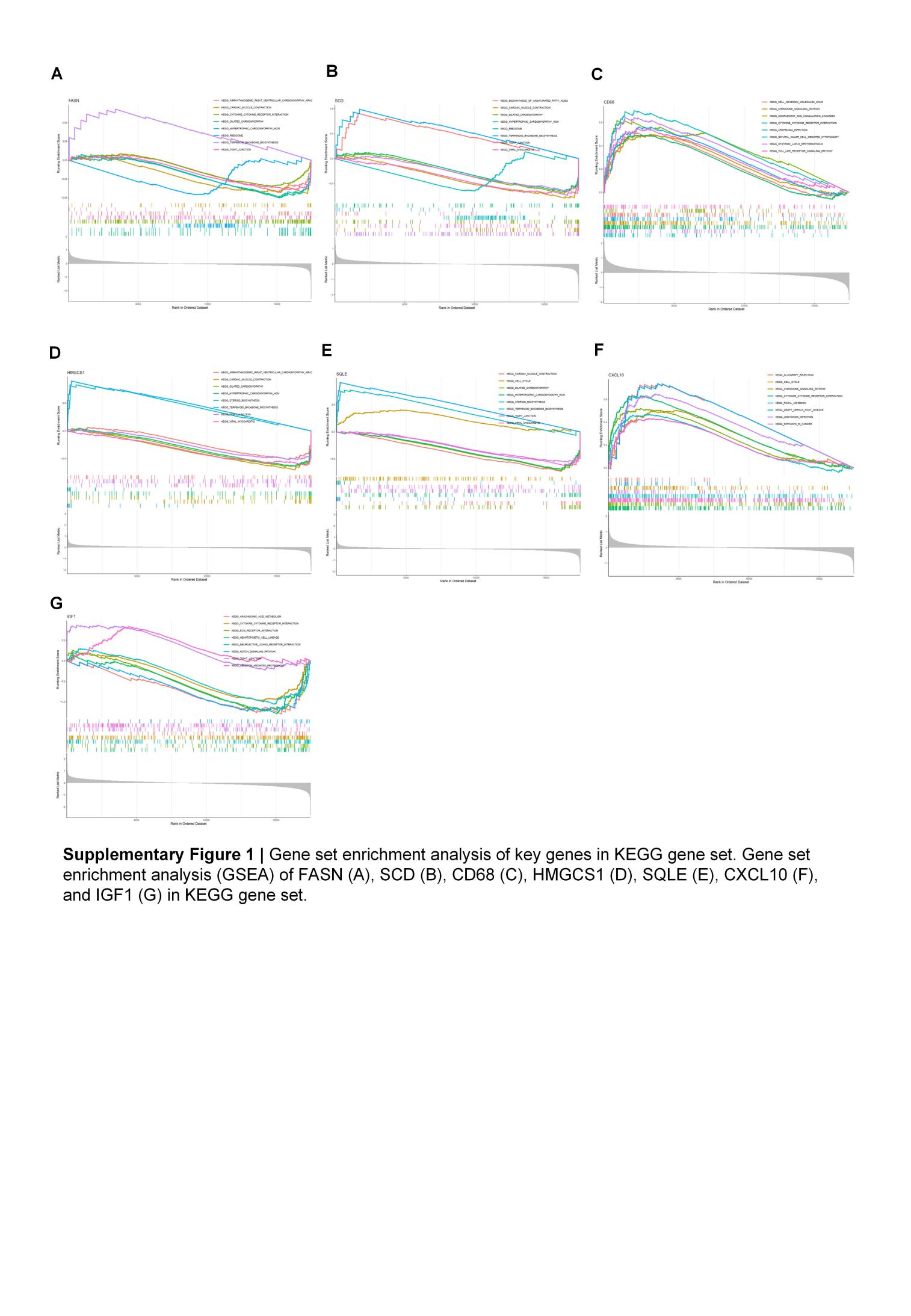
**Supplementary Figure 1.** Gene set enrichment analysis (GSEA) of key genes in KEGG gene set. GSEA of FASN (A), SCD (B), CD68 (C), HMGCS1 (D), SQLE (E), CXCL10 (F), and IGF1 (G) in KEGG gene set.

**
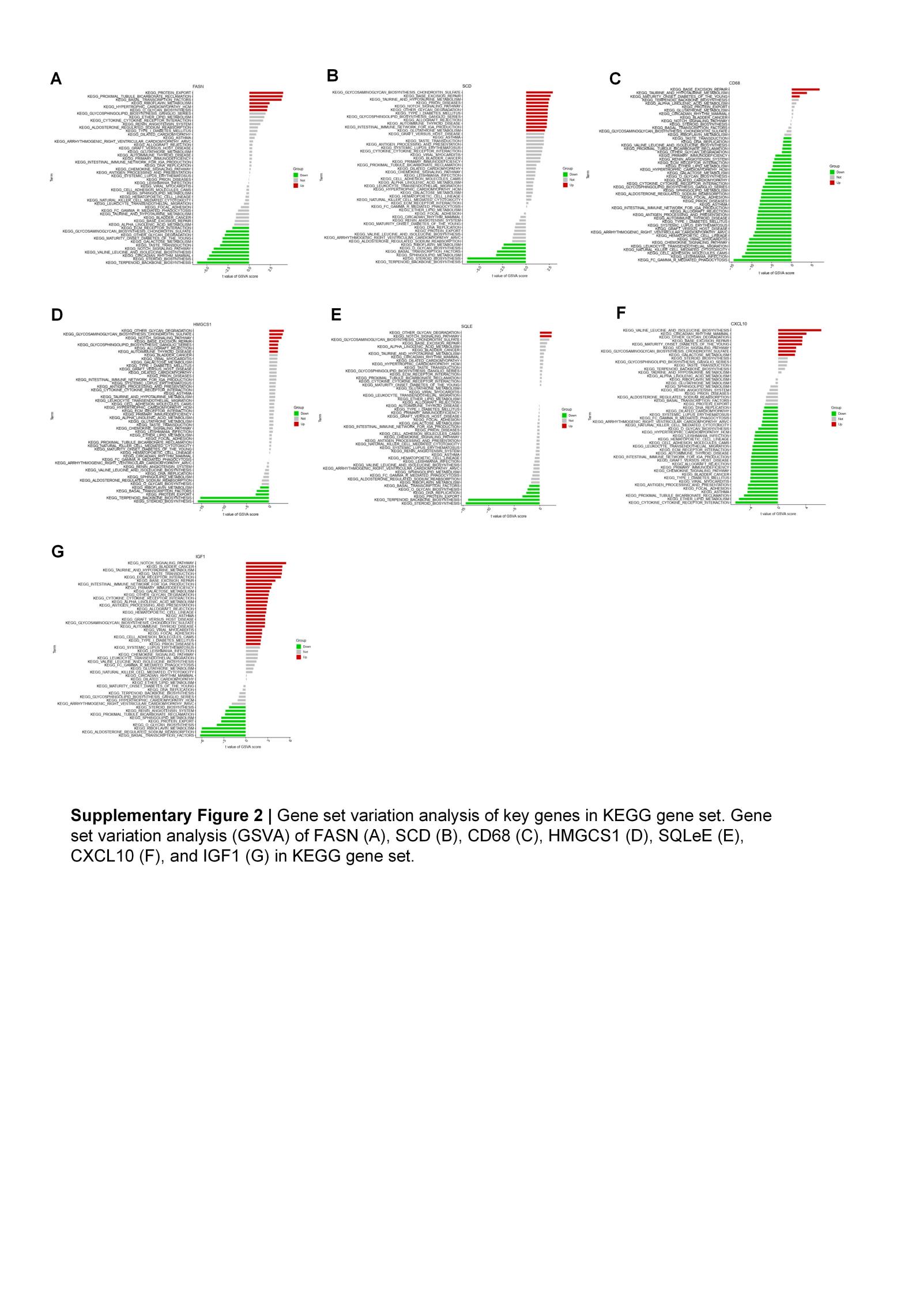
**

**Supplementary Figure 2.** Gene set variation analysis (GSVA) of key genes in KEGG gene set. GSVA of FASN (A), SCD (B), CD68 (C), HMGCS1 (D), SQLeE (E), CXCL10 (F), and IGF1 (G) in KEGG gene set.

## Supplementary Tables

| Gene symbol | Species | Forward Primer(5'-3') | Reverse Primer(5'-3') |
| --- | --- | --- | --- |
| Cd68 | Mouse | CACAGTTTCTCCCACCACAAATG | AAGCCTTTCTTCCACCCTGAATT |
| Hmgcs1 | Mouse | CAGCTCTTGGGATGGACGATATG | CTGAGGTAGCACTGTATGGAGAG |
| Sqle | Mouse | AATTCCGTACCCACTGTCAGAAA | CCCAGTCTCCTTGTCCTTGTATT |
| Cxc10 | Mouse | TTCATCCACCGCTGAGAGACAT | GATAGGCTCGCAGGGATGATTTC |
| Igf1 | Mouse | TCAGCAGCCTTCCAACTCAATTA | CCTGTGGGCTTGTTGAAGTAAA |
| Scd | Mouse | CATCGCCTGCTCTACCCTTTAAA | GTGGTCGTGTAAGAACTGGAGAT |
| Fasn | Mouse | GTTCTAGCCAGCAGAGTCTACAG | GGATACCACCAGAGACCGTTATG |
| β-actin | Mouse | AACAGTCCGCCTAGAAGCAC | CGTTGACATCCGTAAAGACC |

**Supplementary Table 1.** Primer information
